# Mesenteric defect closure versus non-closure after complete mesocolic excision in minimally invasive right colectomy: a retrospective study

**DOI:** 10.1007/s00384-026-05199-3

**Published:** 2026-07-15

**Authors:** Arcangelo Picciariello, Vito Leonardo Pinto, Salvatore Fedele, Fabrizio Aquilino, Vincenzo Papagni, Leonardo Vincenti

**Affiliations:** 1 Department of Experimental Medicine, University of Salento, Lecce, Italy; 2 General Surgery Unit, IRCCS “Saverio De Bellis”, Castellana Grotte, Italy

**Keywords:** Complete mesocolic excision, Colon cancer, Mesenteric defect

## Abstract

**Background:**

The optimal management of the mesenteric defect after minimally invasive right hemicolectomy remains controversial, particularly in the setting of complete mesocolic excision (CME), where a large mesenteric defect is created. This study was aimed at evaluating whether mesenteric defect closure after CME reduces postoperative complications and whether the closure itself introduces additional risks.

**Methods:**

A single-center retrospective study was conducted using a prospectively maintained database of patients with stage II–III right colon cancer who underwent minimally invasive right hemicolectomy with CME between May 2022 and July 2025. Patients were divided into two groups according to whether the mesenteric defect was closed or left open. Demographic, operative, and postoperative outcomes were analyzed, including postoperative ileus (POI), internal herniation, small bowel obstruction, operative time, and length of hospital stay.

**Results:**

A total of 87 patients were included: 46 underwent mesenteric defect closure, and 41 did not. Baseline characteristics were comparable between groups. Postoperative gastrointestinal bleeding occurred in eight patients (9%), while two patients (2.3%) developed anastomotic leakage, one in each group. No symptomatic internal hernias or small bowel obstructions were observed. POI occurred in three patients (3.5%) with similar incidence between groups. Operative time, bowel recovery, harvested lymph nodes, and length of hospital stay did not differ significantly. No complications related to mesenteric defect closure were observed.

**Conclusions:**

Mesenteric defect closure after minimally invasive right hemicolectomy with CME appears safe but does not significantly reduce postoperative morbidity. Given the low incidence of internal herniation, routine closure may not be necessary.

**Supplementary Information:**

The online version contains supplementary material available at https://doi.org/10.1007/s00384-026-05199-3.

## Introduction

The management of mesenteric defects during right hemicolectomy remains a matter of ongoing debate. In open colectomy, mesenteric defects are left open without notable consequences[1]; however, with the widespread adoption of minimally invasive approaches, concerns have emerged that a large, unclosed mesenteric defect may predispose to internal small-bowel herniation, torsion of the anastomosis through the mesenteric defect, and postoperative obstruction. Several case reports have described internal hernias following laparoscopic right hemicolectomy, occasionally requiring urgent reoperation [2, 3]. Nevertheless, large retrospective series have yielded conflicting results: mesenteric defect closure has been associated with significantly lower reoperation rates [4], whereas non-closure resulted in internal hernias requiring surgical intervention in only 0.8% of cases [5]. In the absence of consensus and robust comparative data, the decision to close the mesenteric defect during minimally invasive surgery is often based on surgeon preference rather than standardized guidelines.

The ambiguity surrounding mesenteric defect closure vs. non-closure may be particularly relevant in the setting of complete mesocolic excision (CME), where the mesenteric defect is larger than in resections not involving CME [6]. CME has become an established option for the treatment of stage II–III right-sided colon cancer [7–9], owing to its higher lymph-node yield; however, it inevitably results in a wider mesenteric defect compared to resections not involving CME.

This retrospective comparative study aimed to assess whether mesenteric defect closure after CME is associated with a reduced incidence of postoperative complications and whether the closure itself introduces additional risks.

### Methods

A single-center retrospective study was conducted using a prospectively maintained database of patients affected by right colon cancer at a high-volume tertiary referral center for colorectal cancer treatment.

All human investigations in this study were carried out in full compliance with institutional and national ethical standards, in line with the principles outlined in the Declaration of Helsinki (1964) and its subsequent revisions. Ethical approval was obtained from the Local Ethics Committee (IRCSS Istituto Oncologico “Gabriella Serio,” approval number 2146/CEL). The study methodology and reporting adhered to the STROBE (Strengthening the Reporting of Observational Studies in Epidemiology) guidelines [10].

All patients affected by stage II and III (according to pTNM [11]) right colon cancer undergoing minimally invasive right hemicolectomy with CME between May 2022 and July 2025 were included and analyzed in this study. Right hemicolectomies for benign diseases were excluded as these procedures do not involve formal CME. Because CME radicality inevitably results in a much larger mesenteric defect than standard non-oncological surgery, this restriction allowed for a precise, standardized assessment of this maximal mesenteric defect. Synchronous cancer, open surgery, and transverse colon cancer beyond the hepatic flexure were excluded from the study.

Complete mesocolic excision was performed using a medial-to-lateral or bottom-to-up approach, with en bloc removal of the intact mesocolon containing lymph nodes and dissection along embryological planes to expose the superior mesenteric vein, which served as a key anatomical landmark for central vascular ligation of the feeding vessels [12].

In all cases, an isoperistaltic side-to-side anastomosis was constructed using the Signia™ Powered Stapling System with Tri-Staple™ technology (Medtronic Inc., Minneapolis, MN) and a 60-mm purple cartridge. The enterotomy was closed with a continuous running suture using 3-0 Stratafix™ (Johnson & Johnson MedTech, New Brunswick, NJ), and the closure was reinforced with two additional 3-0 PDS sutures.

The decision to close or leave the mesenteric defect open was at the operating surgeon’s discretion. The non-closure approach was primarily adopted during the initial phase of the study period, whereas routine defect closure was increasingly implemented in the latter phase as a technical preference. No patient-related baseline characteristics influenced the choice of technique.

**Fig. 1:**
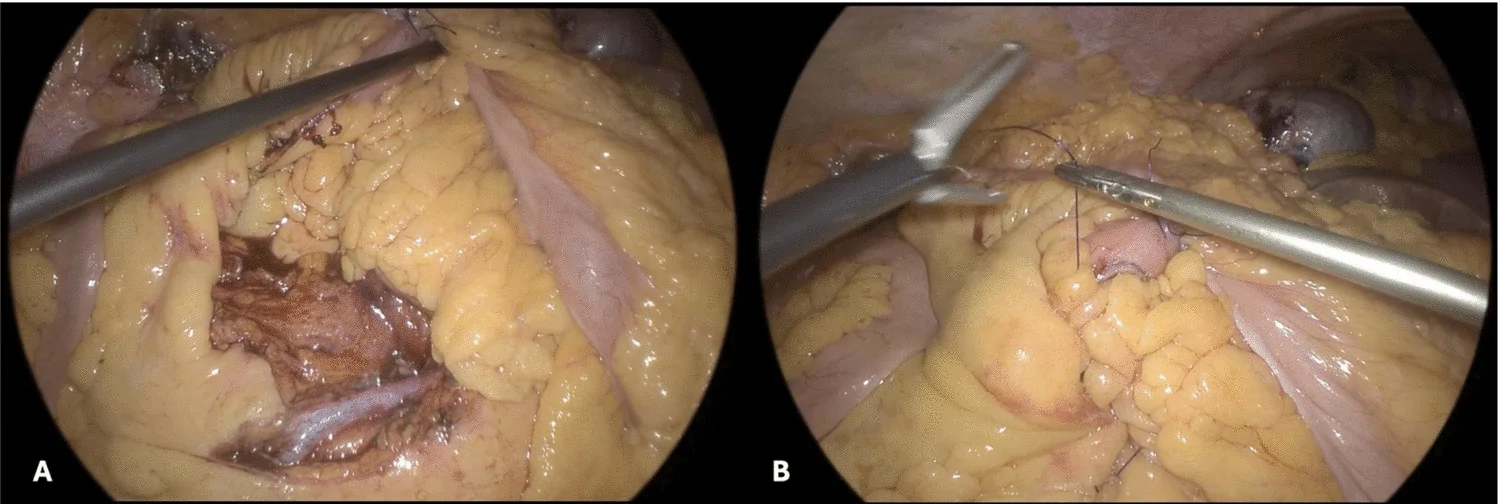
Intraoperative view of the mesenteric defect. **A** Open mesentery, showing the complete exposure of the superior mesenteric vein (SMV). **B** Closed mesentery after the defect repair

When performed, closure of the mesenteric defect was achieved using a continuous running suture with 3-0 Stratafix™ (Johnson & Johnson MedTech, New Brunswick, NJ), initiated at the mesenteric (vascular) margin and continued toward the anastomotic complex (Fig. 1A, B, Supplementary video).

All procedures were performed according to the Enhanced Recovery After Surgery (ERAS) Society recommendations [13].

To ensure strict technical standardization and quality assurance, all surgical procedures (both CME and mesenteric defect closure) were performed by a single senior colorectal surgeon with extensive experience in advanced minimally invasive colorectal surgery (having completed > 400 laparoscopic and robotic right colectomies prior to the study period). This single-operator approach eliminated inter-surgeon technical variability. The surgeon was fully credentialed for CME within our institution. Adherence to the core principles of CME (sharp mesenteric dissection and vascular exposure) was routinely monitored and validated through the pathological assessment of specimen integrity, with all specimens graded as having an intact mesocolic plane.

Demographic and clinical data were retrieved from medical records and Institution Electronic database. Collected variables included age, sex, body mass index (BMI), American Society of Anesthesiologists (ASA) score, history of previous abdominal surgery, minimally invasive approach (laparoscopic vs. robotic), tumor location (cecum, ascending colon, hepatic flexure), operative time, postoperative complications, time to first flatus and first bowel movement, occurrence of postoperative ileus (POI), need for reoperation due to complications, harvested lymph nodes, and length of hospital stay.

POI was defined as a non-mechanical condition preventing oral intake that persisted on or after postoperative day 4 or required the insertion of a nasogastric tube due to clinical worsening after temporary bowel recovery[14]. The most relevant clinical signs were abdominal distension and tenderness and, in third position, absence of normal bowel sounds.

The aim of the study was to evaluate the effects of mesenteric defect closure in preventing postoperative complications, including POI, symptomatic internal herniation, and small bowel obstruction. Secondary outcomes included differences in operative time and length of hospital stay.

#### Statistical analysis

All data were inserted into an Excel database (Microsoft, Redmond, Washington, USA) and analyzed with the Statistical Package for the Social Sciences Windows version 27.0 (SPSS, Chicago, Illinois, USA). Descriptive statistics used were the mean ± standard deviation for continuous parametric variables, median and interquartile range for non-parametric variables, and frequencies for categorical variables. Normality assumptions were demonstrated with histograms and the Shapiro–Wilk test. Analysis of comparison between treatments was conducted with the ANOVA one-way test for continuous parametric variables, Mann–Whitney test for non-parametric variables, and chi-square test for categorical variables (Fisher’s exact test if the counts in cells were inferior to 5). A *p* value less than 0.05 was considered statistically significant.

## Results

During the study period, a total of 87 patients underwent minimally invasive right hemicolectomy with CME for oncologic indications. Out of these, 46 patients underwent closure of the mesenteric defect, while 41 did not (Table 1). The study included 43 males (49.4%) and 44 females (51.6%). The two groups were comparable across the analyzed variables, with no significant differences in age, BMI, ASA grade, history of previous abdominal surgery, tumor location, or operative time.

**Table 1:** Demographics and clinical characteristics of groups (mesenteric closure vs. non-closure)

	Non-closure (*n* = 41)	Closure (*n* = 46)	*p*
Age (years)	75 (65–80)	71 (66–79)	0.772
Sex (males)	18 (43.9%)	25 (54.3%)	0.393
BMI	28 (26–30)	31 (27–32)	0.069
ASA • I • II • III • IV	3 (7.3%) 16 (39.0%) 22 (53.7%) 0 (0%)	1 (2.2%) 23 (50.0%) 19 (41.3%) 3 (6.5%)	0.159
Previous abdominal surgery	11 (26.8%)	10 (21.7%)	0.623
Approach: • Laparoscopic • Robotic	34 (82.9%) 7 (17.1%)	33 (71.7%) 13 (28.3%)	0.308
Tumor location • Cecum • Ascending colon • Hepatic flexure • Transverse colon	7 (17.1%) 20 (48.8%) 13 (31.7%) 1 (2.4%)	15 (32.6%) 22 (47.8%) 9 (19.6%) 0 (0%)	0.179
Duration of surgery (min.)	93 (90–100)	97 (75–140)	0.745
Postop. complications • GI bleeding • Anastomotic leak • Respiratory infections	4 (9.8%) 1 (2.4%) 2 (4.9%)	4 (8.7%) 1 (2.2%) 1(2.2%)	1.000
1 st flatus (postop. day)	2 (1–2)	2 (2–2)	0.440
1 st bowel motion (postop. day)	3 (3–4)	3 (2–4)	0.119
Postop. ileus	1 (2.4%)	2 (4.3%)	0.496
Reoperation	1 (2.4%)	0 (0%)	0.471
Length of stay	5 (5–6)	6 (5–7)	0.233
Harvested lymph nodes	27 (20–34)	26 (20–33)	

Most tumors were located in the ascending colon (48.3%) followed by the cecum (25.3%) and hepatic flexure (25.3%).

Eight patients (9%) experienced postoperative gastrointestinal bleeding, four of whom required blood transfusions. Two patients (2.3%) developed anastomotic leaks, one in each group; one was managed with anastomotic reinforcement and a protective ileostomy, while the other was treated conservatively by antibiotic therapy and drainage monitoring. The former represented the only patient who required reoperation in the series. No adverse events occurred during the mesenteric closure.

No patients experienced symptomatic internal herniation or small bowel obstruction. The incidence of postoperative ileus, as well as the time to first flatus and first bowel movement, was similar between the two groups (Table 1). Postoperative ileus (POI) occurred in 3 patients (3.5%). These patients underwent abdominal radiography, which confirmed the presence of bowel obstruction but did not provide sufficient information to determine its underlying cause. All subjects were conservatively treated by positioning a nasogastric tube.

The median length of hospital stay was 5 days (5–6) in “non-closure” group and 6 days (5–7) in the other group. No differences in harvested lymph nodes were observed among groups. No patients were readmitted within 30 days following discharge.

## Discussion

The optimal management of the mesenteric defect following minimally invasive right hemicolectomy remains controversial, particularly in the setting of CME, where the extent of mesenteric dissection results in a wide defect. In this retrospective comparative study, mesenteric defect closure after CME was not associated with a reduction in postoperative complications, including postoperative ileus, internal herniation, or small bowel obstruction, nor did it increase perioperative morbidity.

Internal herniation through an unclosed mesenteric defect has been reported after laparoscopic right colectomy, mainly in isolated case reports [2, 3]. The concern is amplified in minimally invasive surgery due to reduced postoperative adhesions and increased bowel mobility. However, Cabot et al. specifically evaluated the long-term consequences of leaving the mesenteric defect open and found that, over extended follow-up, the incidence of clinically significant internal hernias remained low, though rare cases could result in late small bowel obstruction requiring reoperation. In fact, small bowel obstruction attributable to the mesenteric defect was observed in 4 patients (0.8%). In two instances, the obstruction resulted from torsion at the anastomotic site passing through the defect, whereas in the remaining two cases it was caused by small bowel herniation through the mesenteric opening [5]. In our cohort of patients, no cases of symptomatic internal hernia or small bowel obstruction were observed in our cohort, regardless of mesenteric defect closure, despite the larger defects inherent to CME.

A case report by Jimi et al. described a late presentation (two months after discharge) of small bowel obstruction caused by internal herniation through a mesenteric defect following laparoscopy-assisted right colectomy [15]. Although our study is limited by the duration of follow-up, no adverse events were recorded, and no patients were readmitted within 30 days of discharge.

Sugiyama et al. reported no significant differences in postoperative outcomes between closure and non-closure following laparoscopic colectomy[4], while Sica et al. demonstrated the technical feasibility and safety of mesenteric defect closure after CME using polymer-ligating clips but no clear evidence of improved clinical outcomes [16].

A concern related to mesenteric closure is the potential increase in operative complexity, operative time, or risk of ischemic or anastomotic complications due to tension on the mesentery. In the present study, defect closure did not result in longer operative times or higher rates of anastomotic leakage, postoperative bleeding, or reoperation, indicating that the procedure can be safely performed when deemed necessary.

In our series, the incidence of POI and bowel recovery parameters were comparable between groups. These findings suggest that POI could be more strongly related to global surgical stress and perioperative management rather than to mesenteric defect closure itself. As expected, mesenteric defect closure did not alter oncological outcomes, with lymph node harvest comparable between groups. This confirms that closure does not compromise the oncological quality of CME, which remains a key component of right-sided colon cancer surgery.

This study has limitations inherent to its retrospective design, including potential selection bias, as the decision to close the mesenteric defect was left to surgeon discretion. The sample size may be insufficient to detect rare events such as late internal herniation, and the follow-up duration may not capture very late presentations.

## Conclusions

Mesenteric defect closure following minimally invasive right hemicolectomy with CME is safe but does not appear to confer a significant clinical benefit in terms of postoperative morbidity. Given the low incidence of internal herniation and POI, routine closure may not be mandatory. Prospective studies are required to further define the role of mesenteric defect closure in this setting.

## Supplementary Information

Below is the link to the electronic supplementary material.

ESM 1 Supplementary video: mesenteric defect closure (MP4 170 MB)

## Data Availability

The data that support the findings of this study are available from the corresponding author upon reasonable request.
